# Cross-cultural adaptation of the Child Perceptions Questionnaire 11–14 (CPQ_11–14_) for the Brazilian Portuguese language

**DOI:** 10.1186/1477-7525-6-2

**Published:** 2008-01-14

**Authors:** Daniela Goursand, Saul M Paiva, Patrícia M Zarzar, Maria L Ramos-Jorge, Gianfilippo M Cornacchia, Isabela A Pordeus, Paul J Allison

**Affiliations:** 1 Department of Pediatric Dentistry and Orthodontics, Faculty of Dentistry, Federal University of Minas Gerais – Av. Antônio Carlos 6627, Belo Horizonte, MG, 31270-901, Brazil; 2 Faculty of Dentistry, McGill University, 3640 University Street, Montreal, QC, H3A 2B2, Canada

## Abstract

**Background:**

Oral-Health-Related Quality of Life (OHRQoL) instruments are being used with increasing frequency in oral health surveys. However, these instruments are not available in all countries or all languages. The availability of cross-culturally valid, multi-lingual versions of instruments is important for epidemiological research. The Child Perceptions Questionnaire 11–14 (CPQ_11–14_) is an OHRQoL instrument that assesses the impact of oral conditions on the quality of life of children and adolescents. The objective of the current study was to carry out the cross-cultural adaptation of CPQ_11–14 _for the Brazilian Portuguese language.

**Methods:**

After translation and cross-cultural adaptation, the CPQ _11–14 _was tested on 160 11-to-14-year-old children who were clinically and radiographically examined for the presence or absence of dental caries. The children were receiving dental care at the Pediatric Dental and Orthodontic clinics of the Federal University of Minas Gerais, Brazil. To test the quality of the translation, 17 children answered the questionnaire. The internal consistency of the instrument was assessed by Cronbach's Alpha Coefficient and the test-retest reliability by Intraclass Correlation Coefficient (ICC).

**Results:**

The mean CPQ_11–14 _score were 24.5 [standard deviation (SD) 18.27] in the group with caries and 12.89 [SD 10.95] in the group without caries. Median scores were 20 and 10 in the groups with and without caries, respectively (*p *< 0.001). Significant associations were identified between caries status and all CPQ domains (*p *< 0.05). Internal reliability was confirmed by a Cronbach's alpha coefficient of 0.86. Test-retest reliability revealed satisfactory reproducibility (ICC = 0.85). The questionnaire proved to be a valid instrument. Construct validity was satisfactory, demonstrating highly significant correlations with global indicators for the total scale and subscales. The CPQ_11–14 _score was able to discriminate between different oral conditions (groups without and with untreated caries).

**Conclusion:**

The present study demonstrated that the CPQ_11–14 _is applicable to children in Brazil. It has satisfactory psychometric properties, but further research is required to evaluate these properties in a population study.

## Background

The clinical indicators used in dentistry have been restricted to the symptoms individuals perceive, such as pain, discomfort and esthetic alterations. It is not yet a common practice for the definition of an oral health policy to consider the impact that such alterations have on the lives of individuals [1]. However, this subject is currently being discussed and instruments that relate health and quality of life are beginning to be employed as supplements to clinical indicators.

A dental survey among 34,550 12-year-old Brazilian school children showed that 69.0% of these children had have dental caries during their lifetime and only 33.0% had access to dental care [2]. These data are important to illustrate the dimension of this social health problem in Brazil. Measuring OHRQoL may add to these powerful data and instruct oral health policy, thus contributing to the definition and prioritization of socially appropriate use of resources.

From a bibliographic survey carried out on the PubMed (National Library of Medicine) indexing database in October 2006 regarding instruments that are specific to dentistry, a combination of descriptors, such as "questionnaire"; "oral health related quality of life", resulted in 127 articles on instruments that assess the relationship between oral health and quality of life. However, most of these were developed for English-speaking adult population. Among the aforementioned 127 articles just 24 concerned children and none concerned Brazilians or the Portuguese language.

The OHRQoL instruments designed to assess the impact of oral conditions on the daily living of children and adolescents are the Child-OIDP (Oral Impacts on Daily Performances) [2], the ECOHIS (Early Childhood Oral Health Impact Scale) [3], the COHQoL (Child Oral Health Quality of Life) [4] and the CPQ (Child Perceptions Questionnaire) [4]. The Child-OIDP has been used with Thai, French and English-speaking children [2,5]. The COHQoL was developed in Canada in the English language [4,6]. The CPQ_11–14 _is a COHQoL questionnaire and is composed of 37 items that assess the repercussions of oral health problems on the quality of life of children between 11 and 14 years of age. Its validity has been demonstrated in English-speaking children in Canada, United Kingdom and New Zealand and, in Arabic in Saudi Arabia [4,7-9].

The lack of instruments of this type in Brazil limits researchers to two alternatives: developing a new instrument or translating, adapting and validating an existing one. The first option has the disadvantages of high cost, prolonged research time and, above all, limitations in terms of comparisons with data from other parts of the world. Thus, the second alternative is more economic, efficient and practical.

The translation and cultural adaptation of instruments is an internationally recognized method [10-14]. Translation consists of obtaining a version that is semantically equivalent to the original. Cross-cultural adaptation is necessary when the instrument is intended for use on a target population that is culturally different from that of the original version. This could require the alteration or removal of items from the original scale. Translation is only one step in the adaptation process. Adaptation may be defined as adapting questionnaires to country- or region-specifics dialects and to cultural context and life-style [15]. A number of instrument translation and cross-cultural adaptation methodologies have been proposed [14,16-20]. One of these methods is a universalist model for the equivalence and adaptation of instruments that relate to health and quality of life [19]. This method consists of six steps: conceptual, item, semantic, operational, measurement and functional equivalence. Following these steps, the adaptation of any health and quality of life instrument can be accomplished.

The aim of the present study was to carry out the cross-cultural adaptation of the CPQ_11–14 _to the Brazilian Portuguese language and to test its reliability and validity.

## Methods

### Description of the Child Perceptions Questionnaire 11–14 (CPQ _11–14_)

The CPQ_11–14 _is a specific questionnaire for assessing the impact of oral health conditions on the quality of life of 11 to 14-year-old children [4]. The items address the frequency of events in the previous three months. It is structurally composed of 37 items distributed among 4 domains: oral symptoms (6 questions), functional limitation (10 questions), emotional well-being (9 questions) and social well-being (12 questions). A 5-point Likert scale is used, with the following options: 'Never' = 0; 'Once/twice' = 1; 'Sometimes' = 2; 'Often' = 3; and 'Every day/almost every day' = 4. The CPQ_11–14 _scores are computed by summing all of the item scores. Scores for each of the four domains can also be computed. Since there were 37 questions, the final score can vary from 0 to 148, for which a higher score denotes a greater degree of the impact of oral conditions on the quality of life of the respondents.

The authors also designed two questions asking the children for a global rating of their oral health and the extent to which their oral health affected their overall well-being was obtained [4]. These questions are: 'Would you say that the health of your teeth, lips, jaws and mouth is...?' and 'How much does the condition of your teeth, lips, jaws or mouth affect your life overall?'. These global ratings had a five-point response format. The responses were scored as follows: for global rating of oral health, (0) excellent, (1) very good, (2) good, (3) fair and (4) poor; and for overall well-being, (0) not at all, (1) very little, (2) somewhat, (3) a lot and (4) very much.

### Adaptation and translation of the CPQ _11–14_

**Figure 1:**
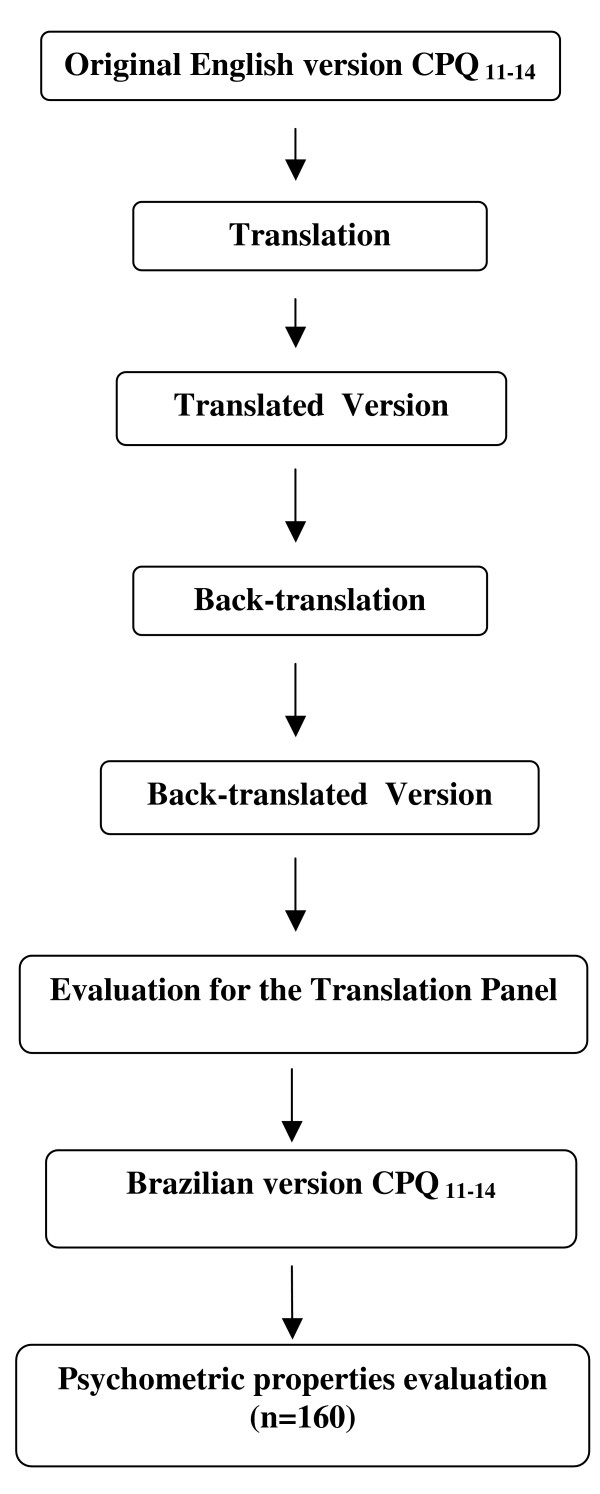
Flow chart of the cross-cultural validation steps.

In order to measure the OHRQoL of children in Brazil, the index needed to be subjected to translation and cross-cultural adaptation in Brazil [19,20]. Based on standard recommendations, two initial translations were made independently by two translators (a Brazilian fluent in the English language and a native English-speaker fluent in Portuguese) with experience in health questionnaire translation. All options were reviewed during consensus meeting in which translation choices and cross-cultural adaptations were made. The translation panel for this meeting consisted of researchers, two translators and three dentists, all fluent in both Portuguese and English. For the determination of conceptual and item equivalence, a group of specialists evaluated this version and compared it to the original. Attention was given to the meaning of the words in the different languages in order to obtain similar effects on respondents from different cultures. An effort was made to identify possible difficulties in understanding the questionnaire. A synthesis-version was developed as a result of this process. The steps of this process are presented in a flow chart (Figure 1).

This draft of the Brazilian version of the CPQ_11–14 _was then pilot-tested on a convenience sample of 37 children. Modifications were made according to the comments made by the children in order to clarify the content of the questionnaire. The children individually suggested the substitution of a number of words and expressions for synonyms in order to facilitate comprehension.

In order to check the translation, this final version was then translated back into English by two native English-speaking individuals who were not previously involved in the study. These two back-translated English versions proved nearly identical. To determine semantic equivalence, a group composed of three dental surgeons fluent in both languages and with no prior knowledge of the study compared the back-translated English version with the original English version. The aim of this step was to achieve a "similar effect" on respondents who speak two languages (English and Portuguese).

The option was made to administer the instrument as an interview in order to reduce losses stemming from self-application and avoid the influence of parents in their children's responses. Structures, instructions, mode of administration and measurement methods were similar to the original English version of CPQ_11–14_. Functional equivalence (the combined effect of assessing conceptual, item, semantic, operational and measurement equivalence) was assessed by a group of specialists with regard to the behavior of the instrument and the possibility of comparisons to studies conducted in different cultures.

### Assessment of validity and reliability of the Brazilian version of the CPQ_11–14_

The study was conducted in Belo Horizonte, capital city of the state of Minas Gerais, Brazil. The city is situated in the central southern region of the state and has 2,238,526 inhabitants.

Data were collected from interviews with 160 children of both genders between 11 and 14 years of age and 83 of these completed the CPQ_11–14_two times and provided data for the assessment of test-retest reliability. They were recruited from pediatric dentistry clinic at the Faculty of Dentistry of the Federal University of Minas Gerais, Brazil. Only subjects who were undergoing dental treatment and were intellectually and physically capable of responding to the questionnaire were included in the study. Parents/guardians and children read and signed an informed consent form prior to participation in the study. The study was approved by the Human Research Ethics Committee of the Federal University of Minas Gerais.

The 160 children completed the Brazilian version of the CPQ_11–14 _questionnaire in the waiting room at the clinic in face-to-face interviews conducted by a trained research assistant. Another investigator reviewed the children's medical records to establish their medical and dental condition on the day of recruitment. All children had current bitewing and panoramic radiographs, dental charts, treatment plans and medical histories so caries status was quantified. The children were separated into two groups: Group 1- included children who either had no untreated caries and/or had completed restorative treatment at least three months earlier and; Group 2- included children who presented untreated caries in one or more teeth, as assessed through clinical and/or radiographic exams. All children were examined by a single dentist/investigator who was previously trained and calibrated (Kappa intra-agreement = 0.90) for the clinical and radiographic diagnosis of dental caries.

The SPSS software program (version 12.0. SPSS Inc., Chicago, IL, USA) was used for the data analysis. Information was codified in a databank. Initially descriptive analyses were performed (average, standard deviation, analysis of total and individual domain scores of the CPQ_11–14_) to generate total and domain CPQ_11–14 _scores for each participant.

Internal consistency of the Brazilian CPQ_11–14 _was assessed using Cronbach's alpha, inter-item and item-total correlation coefficients. Test-retest reliability was assessed by calculating the intraclass correlation coefficient (ICC) with a two-way random effects model for the CPQ_11–14 _score using the data from 83 children who were interviewed a second time by the same investigator 3 weeks following the first interview. To test the construct validity of the Brazilian CPQ_11–14_, associations between the scores of each domain, global oral indicators and overall well-being were analyzed using Spearman's correlation coefficient.

Discriminant validity was tested by comparing the average CPQ_11–14 _scores between the clinical groups studied (group 1 with caries and group 2 without caries). As the CPQ_11–14 _scores were not normally distributed, the non-parametric Mann-Whitney test was used to evaluate the difference in mean scores between the two groups. The level of significance was set at 0.05.

## Results

Of the 184 children initially selected, 24 children had undergone restorative treatment in the previous three months and so were excluded. Thus, the study population consisted of 160 children that received a dental examination and were interviewed using the CPQ_11–14_. Of these, 83 were interviewed a second time three weeks afterward for the test-retest reliability assessment. Gender was evenly distributed. A total of 114 children (71.2%) had no untreated caries and 46 (28.8%) had untreated caries. Average age was 12 years (SD = 1.03), distributed as follow: 40.6% were 11 years old, 28.1% were 12 years old, 20.0% were 13 years old and 11.3% were 14 years-old.

The scores for the total scale in the study population ranged from 0 to 88, with a mean of 16.23 (SD = 14.40). A total of 86.3% of the children reported experiencing oral symptoms in the previous 3 months; 80.0% reported social impacts; 75.0% reported functional limitations and 65.7% reported emotional impacts.

### Reliability

Cronbach's alpha was 0.86 for the total scale and ranged from 0.52 for oral symptoms to 0.86 for emotional well-being, indicating acceptable to good internal consistency (Table 1). Test-retest reliability was assessed using the intraclass correlation coefficient, which was 0.85 for the total scale, 0.49 for oral symptoms, 0.66 for functional limitations, 0.85 for emotional well-being and 0.63 for social well-being (Table 1).

**Table 1:** Reliability statistics for total scale and subscales (n = 83)

Variable	Number of items	Cronbach's alpha	Intraclass correlation coefficient (95% CI)*
Total scale	37	0.86	0.85 (0.82–0.88)
*Subscales*			
Oral symptoms	6	0.52	0.49 (0.35–0.61)
Functional limitations	10	0.69	0.66 (0.58–0.74)
Emotional well-being	9	0.86	0.85 (0.81–0.88)
Social well-being	12	0.66	0.63 (0.53–0.71)

* Two-way random effects model: *p *< 0.001 for all values

### Construct validity

The correlations between the global ratings (overall well-being and oral health) and the total scale (r = 0.26 and 0.38), oral symptoms subscale (r = 0.25 and 0.22), functional limitations subscale (r = 0.19 and 0.35) and emotional well-being subscale (r = 0.36 and 0.33), were mediocre but statistically highly significant. Social well-being subscale was only significantly associated with the global rating of overall well-being (r = 0.21), but not oral health (r = 0.08) (Table 2).

**Table 2:** Construct validity: rank correlations between total scale and subscale scores, and global rating of oral health and overall well-being (n = 160).

	Global rating			
	
	Oral health		Overall well-being	
	r*	*p*-value	r*	*p*-value
Total scale	0.264	0.001	0.382	< 0.001
*Subscales*				
Oral symptoms	0.249	0.002	0.219	0.005
Functional limitations	0.191	0.015	0.352	< 0.001
Emotional well-being	0.356	< 0.001	0.329	< 0.001
Social well-being	0.081	0.308	0.210	0.008

*Spearman's correlation coefficient

### Discriminant validity

There was a significant difference in mean scores for the total, oral symptoms, functional limitations and social well-being between the children without untreated caries (Group 1) and those with untreated caries (Group 2) (Table 3).

**Table 3:** Discriminant validity: overall and subscale scores for children without untreated caries (Group 1) and with untreated caries in one or more teeth (Group 2).

	**Group 1 (n = 114)**		**Group 2 (n = 46)**		*p*-value*
		
	mean ± SD	median	mean ± SD	Median	
Total scale	12.89 ± 10.95	10.00	24.50 ± 18.27	20.00	< 0.001
Subscales					
Oral symptoms	3.49 ± 2.89	3.00	4.74 ± 3.46	4.00	0.035
Functional limitations	2.92 ± 3.54	2.00	6.13 ± 5.65	5.50	< 0.001
Emotional well-being	3.04 ± 4.57	1.00	7.57 ± 7.38	5.00	< 0.001
Social well-being	3.42 ± 3.82	2.00	6.04 ± 5.61	5.00	0.004

* Mann-Whitney Test

## Discussion

Studies assessing the repercussion of oral disorders on the quality of life of individuals have been conducted since the 1980s [21]. However, most of the instruments used have been developed in English-speaking countries [15]. In order to use them with a non English-speaking population these instruments need to be translated, adapted and validated. This process should follow internationally accepted procedures to ensure the resulting new language versions of the questionnaires are valid and can be used in international comparative studies [22].

The Brazilian Portuguese version of the CPQ_11–14 _exhibited acceptable validity and reliability, thus indicating its use for child populations of similar ages in Brazil. The process of translation and cross-cultural adaptation was carefully conducted following the criteria of Herdman et al. (1998) [19] and resulted in a back-translated version that was very similar to the original, thus highlighting the suitability of the Brazilian Portuguese version of the instrument. Test-retest reliability was confirmed by the ICC (0.85) for the total scale. Cronbach's alpha coefficient was 0.86 for the total scale, indicating adequate internal reliability, as reliability of 0.5 or above is considered acceptable [23,24]. For the domains, the coefficient ranged from 0.52 for 'oral symptoms' to 0.86 for 'Emotional well-being'. A similar result was observed in the Canadian pedodontic patients, being the lowest (α = 0.64) and the highest (α = 0.86) coefficients verified in the same subscales [4].

We chose to administer the questionnaire as an interview in order to avoid the possibility of children soliciting help from their parents when having difficulty understanding the questions [7]. However, to allow the assessment of OHRQoL on a wider range of children, the use of a self-completed questionnaire is preferable in population studies due to its lower cost. Therefore, it would be interesting to compare the effect of different modes of administration on the validity and reliability of the questionnaire in this patient population [7]. The items of the CPQ_11–14 _are 'negatively worded'. Items such as 'How often in the past three months have you been unhappy' were characterized as 'negatively worded'. A recent study concluded that items presented in a negative form are better for assessing OHRQoL than items expressed in a positive form, either to reduce response set or assess positive oral health [25].

The present study showed that the OHRQoL measure used was able to discriminate between children without and with untreated dental caries. Individuals with untreated caries had higher average total and subscale scores than individuals without untreated caries (*p *< 0.05). Exact comparison between the results of the Brazilian, Saudi Arabian [7] and Canadian [4] studies was not possible due to the use of different indices for evaluating and analyzing caries status. As with the present study, however, the other two studies demonstrated strong associations between the caries experience and scale scores. In the Canadian study, a strong correlation was observed in pedodontic patients between the number of decayed tooth surfaces and overall scale. In the Saudi Arabian study, a relationship was only demonstrated between caries experience and the oral symptoms scale. On the other hand, in a study performed with 19-years-olds, the CPQ_11–14 _was not able to discriminate between Swedish individuals with high and no caries risk experience [26]. We have to point that this instrument was originally developed for use with children between 11 and 14 years of age and older adolescents are capable of handling situations differently from the younger children previously studied.

Similar to the Canadian study, the global indicator of overall well-being in our Brazilian study correlated with all the domains as well [4]. In the Saudi Arabian study, this indicator was not correlated with social well-being [7]. Nevertheless, the Arabic version of CPQ_11–14 _was presented as valid for its use in Saudi Arabia.

The Portuguese-language translation of the CPQ_11–14 _proved valid and reliable for its use on Brazilian children. However, to allow assessment of OHRQoL of a wider range of children, future studies aimed at validating the shorter and simpler version of the scale [6] should be encouraged and administered in a population study.

## Conclusion

This study provides evidence supporting the cross-cultural validity of a Brazilian Portuguese version of CPQ_11–14 _that can be recommended as an OHRQoL measurement for Brazilian children from 11–14 years.

## Abbreviations

OHRQoL: Oral-Health-Related Quality of Life; CPQ: Child Perceptions Questionnaire; OIDP: Oral Impacts on Daily Performances; ECOHIS: Early Childhood Oral Health Impact Scale; COHQoL: Child Oral Health Quality of Life Questionnaire; ICC: Intraclass Correlation Coefficient; OHIP: Oral Health Impact Profile.

## Competing interests

The author(s) declare that they have no competing interests.

## Authors' contributions

DG, SMP, PMZ, IAP and PJA conceptualized the rationale and designed the study. DG, MLRJ and GMC contributed to the collection of data, statistical analysis and interpretation of the data. DG, MLRJ and SMP conducted the literature review and drafted the manuscript. All authors read and approved the final manuscript.
